# Evaluation of nutritive value of sorghum-based fermented distiller’s dried grain and its effects on growth performance and intestinal functions in broilers

**DOI:** 10.1016/j.psj.2026.107992

**Published:** 2026-10-18

**Authors:** Hailin Huan, Junshu Yan, Yuanlu Tu, Kai Zhang, Gaiqin Wang, Shuaipeng Guo, Honghua Huan, Yumeng Xi

**Affiliations:** a Key Laboratory for Crop and Animal Integrated Farming of Ministry of Agriculture and Rural Affairs, Animal Husbandry Institute, Jiangsu Academy of Agricultural Sciences, 50 Zhongling Street, Nanjing, 210014, China; b Anyou Biological Technology Group Co., Ltd., Suzhou, 215400, China

**Keywords:** Fermented sorghum-based distiller’s dried grain, Broiler, Nutritive value, Apparent digestibility, Intestinal function

## Abstract

This study aimed to evaluate the nutritive value of sorghum-based fermented distiller’s dried grains (FDG) and quantify their dose-response effects on growth performance, nutrient digestibility, intestinal morphology, and cecal microbial communities in broilers. (1) Nutritional composition, amino acid, soluble sugar, and untargeted metabolomics of distiller’s dried grains (DDG) and FDG samples were analyzed. Results showed that FDG had a higher crude protein (CP) and lower crude fiber (CF) content than unfermented DDG (*P* < 0.05). The concentrations of most hydrolyzed amino acids (AA) and soluble sugars were markedly increased in FDG (*P* < 0.05). Untargeted metabolomics identified 169 differential metabolites between DDG and FDG, with FDG enriched in bioactive compounds including “polyphenols and flavonoids”, “nucleosides, nucleotides and analogs” and “organic acids and derivatives,” whereas “alkaloids and derivatives” were downregulated. (2) Furthermore, *in vitro* monogastric simulated digestion and *in vivo* total excreta collection assays using 21-day-old broilers demonstrated that fermentation significantly improved apparent CP and CF digestibility. (3) For the feeding trial, a total of 540 1‑day‑old male AA broilers with an average initial body weight of 38.79 ± 0.14 g were randomly allocated into six dietary treatments with 6 replicates per treatment and 15 birds per replicate, and the trial lasted for 42 d. The dietary treatments included a basal diet supplemented with 0% (F0), 2.5% (F2.5), 5% (F5), 10% (F10), 15% (F15), or 20% (F20) FDG, respectively. Growth performance results showed that F2.5-F5 groups maintained growth performance comparable to that of the control group, whereas 20% FDG supplementation markedly impaired F/G (P < 0.05) across all growth phases. Serum biochemistry indicated that FDG supplementation up to 15% had no adverse effects on liver and kidney function, but 20% FDG increased alanine aminotransferase and aspartate aminotransferase activities (*P* < 0.05). Intestinal morphology analysis revealed that 20% FDG deepened jejunal crypt depth and reduced villus height/crypt depth (V/C) ratio (*P* < 0.05), whereas F0-F15 groups maintained normal intestinal architecture. Cecal microbiota analysis showed that 2.5%-5% FDG increased microbial diversity (Shannon index) and enriched beneficial genera (*Butyricicoccus, Lactobacillus, Faecalibacterium*), whereas 20% FDG promoted the proliferation of *Escherichia-Shigella* (*P* < 0.05). In conclusion, fermentation effectively enhances the nutritive value of sorghum-based DDG. Supplementation with 2.5%–5% FDG maintained growth performance comparable to that of the control while improving gut health; 10% FDG can be used for soybean meal replacement in practical production without compromising performance; and the maximum safe inclusion level is 15% in broiler diets.

## Introduction

Global supplies of feed-grade energy and protein ingredients are tightening, mainly because of climate change in major producing regions, shipping disruptions, and strong demand from the livestock farming sectors, driven corn and soybean meal (SBM) prices to record highs (FAO, 2023). Thus, it is necessary to develop effective technologies to improve the bioavailability of feed resources and explore novel feed resources with high nutritional value (Li et al., 2022). In this regard, the byproduct of liquor production is a better candidate as a feed resource. China produces more than 12 million tons of liquor and over 30 million tons of by-products from solid-state alcohol distillation annually. As available by-products, these sorghum-based distiller’s dried grains (DDG) contain 140–180 g crude protein (CP) kg⁻¹ dry matter (DM), 50–90 g crude fat kg⁻¹ DM, 180–250 g crude fiber (CF) kg⁻¹ DM, appreciable phosphorus and minerals (Tian et al., 2017; Chen et al., 2025). However, the application of distiller’s grains in poultry production has certain limitations. High fiber and the presence of anti-nutritional factors have restricted its supplementation to lower than 25 g kg⁻¹ in broiler diets (Ma et al., 2026). Therefore, the development of effective technologies to improve the bioavailability of sorghum-based DDG is crucial for enhancing its feed value.

Solid-state fermentation (SSF) is commonly used to improve the nutritional value of unconventional feedstuffs. Relative to untreated byproducts, SSF degrades lignocellulose, enriches small peptides and nucleotides, and generates a rich repertoire of bioactive metabolites, including yeast autolysates, probiotic biomass, immune-modulating polysaccharides, and other physiologically active compounds (Tian et al., 2017). These biochemical changes improve CP and fiber digestibility (Li et al., 2022), thereby increasing the nutritive value of the fermented residue. SSF also produces a spectrum of small-molecule metabolites and bioactive compounds in fermented distiller’s dried grains (FDG), which may modulate performance, gut function, and immunity in animals (Roper et al., 2019). However, the composition and content of these fermentation-derived molecules in FDG remain poorly understood. Moreover, studies on sorghum-based FDG in poultry production are limited because most poultry studies have mainly focused on maize- or wheat-based DDG (Meloche et al., 2013; Jacobs and Parsons, 2013). Consequently, the present study assessed the nutritive value and apparent digestibility of nutrients of FDG and quantified its dose-response effects on growth performance, serum biochemistry, intestinal morphology, and cecal microbial communities in broilers.

## Materials and methods

### Animal ethics

The study was approved by the Research Committee of the Jiangsu Academy of Agricultural Sciences and conducted according to the Regulations of the Administration of Affairs Concerning Experimental Animals (Order No. 63 of the Jiangsu Academy of Agricultural Science on July 8, 2014).

### Preparation of FDG

DDG and FDG originated from the dry distiller stream of Yanghe Distillery Co., Ltd. (Suqian, Jiangsu, China). For FDG production, DDG was inoculated with 0.3% (w/w) *Saccharomyces cerevisiae* (5 × 10⁸ CFU g⁻¹, provided by Hubei Gaosheng Bio-Technology Co., Ltd., Wuhan, China), adjusted to 48%−50% moisture, and subjected to SSF for 48-72 h at 25-50 °C under airtight conditions. After fermentation, the product was dried at 60 °C to <10% moisture, milled to pass through a 40-mesh sieve, heat-sealed in polyethylene pouches, and stored at 4°C until analysis. The DDG and FDG samples for the nutritional composition analysis, randomly acquired from five locations, were mixed and analyzed in triplicate.

Crude protein level was determined using the Kjeldahl method according to China National Standard (2018) (GB/T, 6432-2018, 2018). Ether extract (EE) level was determined according to China National Standard (2006) (GB/T, 6433-2006, 2006). Crude fiber was measured using an ANKOM Automated 2000i Fiber Analyzer (ANKOM Technology, NY, USA). Calcium level was determined using the ethylene diamine tetraacetic acid (EDTA) disodium complexometric titration method according to China National Standard (2018) (GB/T, 6436-2018, 2018). Total phosphorus level was determined by spectrophotometry according to China National Standard (2018) (GB/T, 6437-2018, 2018).

### Determination of soluble sugars

Soluble sugars were quantified with minor modifications. Briefly, 10–100 mg of sample was weighed and extracted with 700 µL of 80% (v/v) ethanol at 50°C for 2 h. After addition of 700 µL ultrapure water, the suspension was vortex-mixed and centrifuged at 10,000 × *g* for 3 min at 4°C. The supernatant was diluted to an appropriate concentration with ultrapure water, and filtered through a 0.22 µm nylon membrane before measurement. Chromatographic separation was performed using a Dionex ICS-5000 ion chromatography system (Thermo Fisher Scientific, USA) equipped with an electrochemical detector. A CarboPac™ PA1 analytical column (250 mm × 4.0 mm i.d., 10 µm particle size) was used at 30°C. The mobile phases consisted of ultrapure water and 100 mM sodium hydroxide delivered at 1.0 mL/min using a gradient program. The injection volume was 10 µL. Data acquisition and peak integration were carried out with Chromeleon™ 7.2 software (Thermo Fisher Scientific, USA).

### Determination of amino acid composition

The samples (100 mg) were hydrolyzed under vacuum with 6 M HCl containing 0.1% phenol for 24 h at 110°C. After filtration and dilution, the hydrolysates were analyzed using a high-speed automatic Amino acid (AA) analyzer (L-8900, Hitachi Ltd., Tokyo, Japan) equipped with a lithium-cation-exchange column (4.6 × 150 mm). Then, ninhydrin reagent was pumped at 0.25 mL min⁻¹, and the resultant purple (570 nm) or yellow (440 nm for proline) derivatives were monitored using a detector.

### Untargeted metabolite profiling of distiller’s grains

Untargeted metabolite profiling was performed using liquid chromatography-mass spectrometry (LC-MS) to identify metabolites from distiller's grain samples. All procedures were performed by LC-Bio Technologies Co., Ltd. (Hangzhou, Zhejiang, China). Briefly, 100 mg sample was extracted with 1 mL ice-cold methanol/acetonitrile (1:1, v/v), vortexed for 30 s, held at −20°C for 20 min, and centrifuged at 12 000×g for 15 min at 4°C. The supernatant was transferred to an LC vial, and 2 μL was injected for analysis. Chromatographic separation was performed using a Waters ACQUITY UPLC BEH C18 column. Raw files were processed using Xcalibur 4.2 software, and peak detection, retention time alignment, and integration were completed in MetLab (v1.0). Data were log-transformed and Pareto-scaled before multivariate analysis. Principal component analysis (PCA) and orthogonal partial least squares discriminant analysis (OPLS-DA) were conducted using SIMCA 16 software (Umetrics, Sweden). Variable importance in projection(VIP) >1.0 and Student’s *t*-test at *P* < 0.05 were used to identify differential metabolites.

### In vitro and in vivo apparent digestibility

*In vitro* DM and CP disappearance of DDG and FDG were determined using the SDS-III Monogastric Simulated Digestion System (Hunan Zhongben Intelligent Technology Development Co., Ltd., Hunan, China) following the manufacturer’s instructions. According to the method described by Liu et al. (2021), 1.0 g sample was sequentially incubated with simulated gastric fluid (pH 2.0, 4 h, 41°C), proximal intestinal fluid (pH 6.5, 7.5 h, 41°C) and distal intestinal fluid (pH 7.99, 7.5 h, 41°C). The residues were filtered, washed using ethanol and acetone, dried, and analyzed for DM and CP. *In vitro* DM digestibility and CP digestibility were calculated after correcting for the enzyme blanks.

*In vivo* apparent total tract digestibility was determined by total excreta collection (Manyeula et al., 2019). Twenty-four 21-day-old broilers were randomly assigned to three dietary treatments with eight replicates each (one bird per replicate cage). The following diets were used: (1) basal, corn-soybean feed (Table 1) formulated to the broiler nutrient standards (GB/T, 5916-2020, 2020); (2) experimental DDG diet, basal diet in which 20% of the DM was replaced by DDG; and (3) experimental FDG diet, the basal diet in which 20% of the DM was replaced by FDG. A 20% inclusion level was selected for the digestibility assay to maximize the measurable differences in nutrient digestibility between DDG and FDG and to improve the reliability of the apparent digestibility coefficient (ADC) calculation, because a higher inclusion level amplifies the difference in nutrient output between the experimental and basal diets and reduces the relative error of the difference method (Bertechini et al., 2020). After a 7-day adaptation period (days 1–7), total excreta were collected daily for the subsequent 72 h (days 8–10), immediately fixed using nitrogen with 10% sulfuric acid, mixed per replicate, and frozen at −20°C until analyzes. Because total excreta in poultry contain both fecal and urinary nitrogen, the apparent CP digestibility reported here was calculated from the total nitrogen content of the diet and of the total excreta (i.e., apparent nitrogen retention). The feed intake was recorded daily for each replicate. The ADC of the raw materials was calculated as follows:

(1) ADC=1−−(nutrientoutput/nutrientintake)

(2) RawmaterialADC=(experimentaldietADC−basaldietADC)/20%+basaldietADC

where nutrient output is the total nutrients excreted during the 72 h collection period, and nutrient intake is the total nutrients consumed during the same period.

**Table 1:** Ingredient and nutrient composition of the based diet in the metabolism test (DM%).

Items	Content
Ingredients 1, %	
Corn	60.28
Soybean meal	25.85
Corn protein meal	3.00
Soybean oil	2.91
NaCl	0.22
Limestone	0.98
CaHPO_4_	1.98
*L*-Lys·HCl	0.34
*DL*-Met	0.09
*L*-Thr	0.09
*L*-Arg	0.03
*L*-IIe	0.03
Zeolite powder	3.20
Premix 2	1.00
Total	100.00
Nutrient levels 3	
Poultry ME, kcal/kg	3050
CP, %	18.00
OM, %	82.53
Calcium, %	0.88
Phosphorus, %	0.67
Available phosphorus. %	0.39
CF, %	4.50

1 FDG: fermented distillers dried grains; ME: metabolic energy; CP: crude protein; OM: organic matter; DM: dry matter.

2 The premix provided the following per kilogram of diet: VA 3560 IU, VB_1_ 2.4 mg, VB_2_ 4.0 mg, VD_3_ 603 IU, VE 20 mg, VB_12_ 0.01 mg, niacin 16.2 mg, pantothenic acid 10 mg, pyridoxine 3.5 mg, biotin 0.15 mg, folic acid 0.55 mg, choline chloride 750 mg, Cu 18.8 mg, Fe 80 mg, Mn 60 mg, Zn 63 mg, I 0.60 mg, Se 0.19 mg.

3 The poultry ME, available phosphorus, and CF were calculated values. The CP, calcium, and phosphorus were analyzed values. Dietary OM level was calculated by subtracting the crude ash level from the dry matter level in the diet.

### Animals and experimental design

A total of 540 1-day-old male AA broilers with an average initial body weight of 38.79 ± 0.14 g were obtained from Anyou Biological Technology Group Co., Ltd. (Jiangsu, China). The birds were randomly allocated to six treatments in a completely randomized design, which were then supplemented with 0% (F0), 2.5% (F2.5), 5% (F5), 10% (F10), 15% (F15), or 20% (F20) FDG. Each treatment comprised six replicate pens (n = 6) with 15 birds per pen. The basal diet was formulated to the formula feeds for layers and broilers (GB/T, 5916-2020, 2020) and the National Research Council (1994) recommendations. The compositions of the experimental diets are shown in Table 2. Metabolizable energy levels were calculated based on the proportion of each ingredient in the feed formulation and the nutritive values of the ingredients listed in the tables of feed ingredients nutritive values in China (2019) and a commercial feed ingredient database from Haid Group. The dietary CP, calcium, total phosphorus levels were determined using the same methods as described in Section (Preparation of FDG). Dietary organic matter (OM) level was calculated by subtracting the crude ash level from the dry matter level in the diet.

**Table 2:** Ingredient and nutrient composition of diets with different FDG levels supplement (DM%).

Items	1 to 21 days of age						22 to 42 days of age					
	F0	F2.5	F5	F10	F15	F20	F0	F2.5	F5	F10	F15	F20
Ingredients 1, %												
Corn	55.86	53.51	51.18	46.55	41.84	36.60	54.86	52.31	49.7	44.63	39.63	34.58
Soybean meal	31.25	30.70	30.10	28.90	27.75	27.11	33.30	32.90	32.55	31.70	30.80	29.90
Corn protein meal	4.00	4.00	4.00	4.00	4.00	4.00	2.00	2.00	2.00	2.00	2.00	2.00
cottonseed meal	3.00	3.00	3.00	3.00	3.00	3.00	-	-	-	-	-	-
hydrolyzed feather meal	-	-	-	-	-	-	1.00	1.00	1.00	1.00	1.00	1.00
Soybean oil	1.20	1.60	2.05	2.92	3.80	4.75	4.90	5.35	5.82	6.75	7.65	8.60
Limestone	1.42	1.38	1.34	1.2	1.09	0.98	0.89	0.87	0.85	0.81	0.77	0.73
CaHPO_4_	1.30	1.32	1.32	1.35	1.37	1.39	1.08	1.08	1.07	1.07	1.07	1.07
*L*-Lys·HCl	0.46	0.48	0.49	0.53	0.56	0.57	0.43	0.44	0.45	0.47	0.48	0.50
*DL*-Met	0.30	0.30	0.30	0.31	0.31	0.31	0.31	0.31	0.31	0.30	0.30	0.30
*L*-Thr	0.11	0.11	0.12	0.13	0.14	0.14	0.11	0.11	0.11	0.12	0.13	0.13
*L*-Arg	-	-	-	0.01	0.04	0.05	0.02	0.03	0.04	0.05	0.07	0.09
Premix 2	1.10	1.10	1.10	1.10	1.10	1.10	1.10	1.10	1.10	1.10	1.10	1.10
FDG 1	-	2.50	5.00	10.00	15.00	20.00	-	2.50	5.00	10.00	15.00	20.00
Total	100.00	100.00	100.00	100.00	100.00	100.00	100.00	100.00	100.00	100.00	100.00	100.00
Nutrient levels 3												
Poultry ME, kcal/kg	3000	3000	3000	3000	3000	3000	3150	3150	3150	3150	3150	3150
CP,%	22.09	21.73	21.88	21.93	21.47	21.92	20.25	20.04	20.98	20.11	20.98	20.21
OM, %	82.89	82.52	82.93	83.04	82.88	82.97	83.04	83.10	82.98	83.15	82.96	82.51
Calcium, %	0.91	0.90	0.90	0.92	0.91	0.92	0.91	0.93	0.92	0.94	0.93	0.94
Phosphorus, %	0.61	0.63	0.61	0.61	0.63	0.61	0.60	0.60	0.61	0.60	0.60	0.61
Available phosphorus. %	0.30	0.30	0.30	0.30	0.30	0.30	0.30	0.30	0.30	0.30	0.30	0.30
CF, %	3.37	3.55	4.02	4.26	4.31	4.57	3.93	4.01	4.09	4.64	5.09	5.44
NDF, %	8.51	9.22	9.93	10.35	11.76	12.20	8.70	9.41	10.12	11.05	11.97	12.39
ADF, %	3.63	3.90	4.16	4.70	5.23	5.80	4.12	4.39	4.67	5.23	5.77	6.32
SID-Lys, %	1.28	1.28	1.28	1.28	1.28	1.28	1.02	1.02	1.02	1.02	1.02	1.02
SID-Met, %	0.51	0.51	0.51	0.51	0.51	0.51	0.45	0.45	0.45	0.45	0.45	0.45
SID-Thr, %	0.87	0.87	0.87	0.87	0.87	0.87	0.68	0.68	0.68	0.68	0.68	0.68
SID-Arg, %	1.38	1.38	1.38	1.38	1.38	1.38	1.10	1.10	1.10	1.10	1.10	1.10

1 FDG: fermented distillers dried grains; ME: metabolic energy; CP: crude protein; OM: organic matter; CF: crude fiber; ADF: Acid Detergent Fiber; NDF: Neutral Detergent Fiber.

2 The premix provided the following per kilogram of diet: VA 6000 IU, VB_1_ 3.8 mg, VB_2_ 4.0 mg, VD_3_ 543 IU, VE 40 mg, VB_12_ 0.01 mg, niacin 41.8 mg, pantothenic acid 10 mg, pyridoxine 3.5 mg, biotin 0.15 mg, folic acid 1.5 mg, choline chloride 1000 mg, Cu 18.8 mg, Fe 80 mg, Mn 60 mg, Zn 74 mg, I 0.60 mg, Se 0.11 mg.

3 The poultry ME, available phosphorus were calculated values. The CP, calcium, and phosphorus were analyzed values. Dietary OM level was calculated by subtracting the crude ash level from the dry matter level in the diet.

The broilers were reared in three-tier battery cages in an environmentally controlled room. The temperature was maintained at 30 ± 1°C for the first 3 days and then gradually reduced by 1°C every 3 days until 24°C was reached and was kept constant thereafter. Natural light was continuously supplied for 12 h (6:00 AM to 6:00 PM), and artificial light was supplied for 12 h (6:00 PM to 6:00 AM). Feed and fresh water were available ad libitum throughout the 42-day experiment, with a starter diet (0–3 weeks) and a grower diet (3–6 weeks) prepared for the different treatments. The average daily feed intake (ADFI) and live body weight of the broilers were recorded using an electronic scale (YP60001; Hengji, Shanghai, China) before feeding, and the ADG and feed conversion ratio (F/G) were calculated at the end of the experiment.

At the end of the 42-day experiment, one bird per replicate were randomly selected and slaughtered by cutting carotid arteries. Blood samples were collected, and serum was obtained by centrifugation at 1500 × g for 15 min and stored at −80°C until biochemical analysis. Serum alanine aminotransferase (ALT), aspartate aminotransferase (AST), blood urea nitrogen (BUN), creatinine (Cr), and uric acid (UA) concentrations were determined using a fully automatic biochemical analyzer (AU5800, Beckman Coulter Co., Ltd., Brea, CA) according to the manufacturer’s instructions. Immediately after slaughter, jejunal and ileal segments were collected for morphological analysis, and cecal digesta were collected into sterile tubes and stored at −80°C until 16S rRNA gene sequencing.

### Intestinal morphology

Jejunal and ileal samples were collected for histological analyzes. Samples were washed using ice-cold PBS (pH 7.4), fixed in 4% paraformaldehyde, dehydrated through a graded ethanol series, cleared in xylene, and embedded in paraffin, and sectioned to 3 μm thickness (four slices per broiler). The villus heights and crypt depths of 15 well-oriented villi per segment were measured using a Nikon ECLIPSE 80i light microscope equipped with a computer-assisted morphometric system (Nikon Corporation, Tokyo, Japan). Villus height was measured from the tip of the villus to the villus–crypt junction, and crypt depth from the villus–crypt junction to the base of the crypt as described by Lawrence-Azua et al. (2026).

### 16S rRNA sequencing and bioinformatics

DNA was extracted from the cecum samples using a MicroElute Genomic DNA Kit (D3096-01, Omega Biotek Inc., Norcross, GA, USA), according to the manufacturer’s instructions. Total DNA was eluted in 50 μL elution buffer and stored at −80°C until amplification by PCR, which was performed by LC-Bio Technology Co., Ltd, Hang Zhou, Zhejiang Province, China. Forward 343F (5′-TACGGRAGGCAGCAG-3′) and reverse 798R (5′-AGGGTATCTAATCCT-3′) primers were used to amplify the V3 to V4 regions of bacterial 16S rRNA. Amplicon pools were prepared for sequencing, and the size and quantity of the amplicon library were assessed using an Agilent 2100 Bioanalyzer (Agilent, Santa Clara, CA, USA) and a Library Quantification Kit for Illumina (Kapa Biosciences, Woburn, MA, USA), respectively. Libraries were sequenced using a NovaSeq PE250 platform (Illumina, San Diego, CA, USA). Paired-end reads were assigned to samples based on their unique barcodes and truncated by removing the barcodes and primer sequences. Paired-end reads were merged using FLASH software. Quality filtering of raw reads was performed under specific conditions to obtain high-quality clean tags using fqtrim v0.94. Chimeric sequences were filtered using Vsearch software v2.3.4. The GenBank accession number for the amplicon sequence variants (ASV) is PRJNA1029764. The reads were filtered using Quantitative Insights Into Microbial Ecology 2 (QIIME2) quality filters. After dereplication using DADA2 (QIIME2-dada2), alpha and beta diversity indices were calculated by random normalization of the same sequences. Then, according to the SILVA (release 138) classifier, feature abundance was normalized using the relative abundance of each sample. Alpha diversity was applied to analyze the complexity of species diversity using four indices, including Chao1, Observed species, Shannon, and Simpson. All indices in the samples were calculated using QIIME2. Beta diversity was calculated using QIIME2, and graphs were produced using R software v3.5.2. BLAST was used for sequence alignment, and feature sequences were annotated using the SILVA database for each representative sequence.

### Data analysis

Data concerning the nutritional composition of DDG and FDG were analyzed using independent-samples *t*-tests. The data from the animal experiment were subjected to one-way ANOVA using the general linear model (GLM) procedure in SPSS 18.0 (IBM Corp., Somers, NY, USA). The mathematical model for the statistical analysis was as follows:

$$Y_{ij}=\mu +T_{i}+\varepsilon_{ij};$$

Where *Y _ij_* serves as the dependent variable; *μ* represents the overall mean; *T _i_* represents the fixed effect of dietary FDG treatment; and *ε _ij_* represents the random error. Linear and quadratic regression analyzes were performed to evaluate the dose–response effects of supplementing FDG levels. Linear and quadratic regression model:

$$Y=\beta_{0}+\beta_{1}X+\beta_{2}X^{2}+e$$

Where *Y* = dependent variable; *X* = dietary FDG level; *β*_0_ = intercept; *β*_1_ = linear coefficient; *β*_2_ = quadratic coefficient; *e* = residual error. Tukey’s multiple range test was conducted to separate treatment means when the ANOVA F-test was significant (*P* < 0.05). The differences were considered significant at P < 0.05.

## Results

### Nutritive values of DDG and FDG samples

A systematic nutritive comparison between DDG and FDG samples is presented in Table 3. After fermentation, FDG samples exhibited a significantly higher CP and Ash concentrations than DDG samples (*P* < 0.05), accompanied by a pronounced reduction in CF, ADF and NDF contents (*P* < 0.05). Total P tended to increase in FDG samples (*P* < 0.10), whereas no significant changes were observed in EE or Ca levels (*P* > 0.05). Among the hydrolyzed amino acids (Fig. 1), the total amino-acid content in the FDG samples was markedly higher than that in DDG samples (*P* < 0.05). This enhancement was primarily attributable to the upregulation of aspartic acid (Asp), threonine (Thr), glycine (Gly), alanine (Ala), serine (Ser), methionine (Met), cysteine (Cys), phenylalanine (Phe), lysine (Lys), tyrosine (Tyr), histidine (His), and arginine (Arg) (*P* < 0.05). In contrast, glutamic acid (Glu), isoleucine (Ile), valine (Val), leucine (Leu), and proline (Pro) remained unchanged (P > 0.05; Pro: P = 0.050). Analysis of soluble sugars revealed that the concentrations of trehalose, fucose, glucose, fructose, sucrose, raffinose, and stachyose were significantly higher in FDG samples than in DDG samples (*P* < 0.05), whereas the arabinose content was not significantly affected by fermentation (*P* > 0.05).

**Table 3:** Analysis on the nutritional compositions of DDG and FDG samples (DM%).

Items	Groups		SEM	*P* value
	DDG	FDG		
Conventional nutritive values				
CP %	17.96	21.98	2.882	0.042
Ash, %	6.83	7.46	0.216	0.041
NDF, %	36.10	26.75	1.398	0.006
ADF, %	25.35	18.26	1.112	0.010
EE %	6.09	8.40	0.901	0.110
CF %	19.47	14.87	1.772	0.023
Ca %	0.40	0.36	0.260	0.384
P %	0.29	0.47	0.131	0.060
Hydrolyzed amino acids				
Asp %	0.72	1.29	0.263	0.032
Thr %	0.37	0.69	0.167	0.041
Ser %	0.49	0.85	0.198	0.038
Glu %	2.91	3.81	0.517	0.158
Gly %	0.45	0.74	0.149	0.030
Ala %	0.90	1.40	0.291	0.038
Cys %	0.13	0.25	0.057	0.024
Val %	0.60	0.95	0.184	0.224
Met %	0.09	0.21	0.064	0.031
Ile %	0.43	0.71	0.141	0.118
Leu %	1.07	2.02	0.494	0.730
Tyr %	0.14	0.51	0.165	0.034
Phe %	0.55	0.91	0.192	0.049
Lys %	0.30	0.59	0.157	0.029
His %	0.23	0.44	0.116	0.048
Arg %	0.32	0.69	0.176	0.022
Pro %	1.05	1.50	0.262	0.050
Total	10.76	17.55	3.490	0.029
Soluble sugars				
Tre μg/ml	0.43	4.03	0.149	<0.001
Fuc μg/ml	1.00	8.15	0.523	<0.001
Ara μg/ml	0.51	0.66	0.079	0.133
Glc μg/ml	0.32	0.97	0.050	<0.001
Fru μg/ml	0.00	0.66	0.027	<0.001
Suc μg/ml	2.42	16.41	0.621	<0.001
Raf μg/ml	0.00	3.70	0.134	<0.001
Sta μg/ml	0.00	9.59	0.251	<0.001

DDG: distiller’s dried grains; FDG: fermented distiller’s dried grains; CP: crude protein; EE: ether extract; CF: crude fiber; ASP, aspartic acid; Thr, threonine; Gly, glycine; Ala, alanine; Ser, serine; Glu, glutamic acid; Met, methionine; IIe, isoleucine; Val, valine; Phe, phenylalanine; Lys, lysine; Leu, leucine; Tyr, tyrosine; Pro, proline; His, histidine; Arg, arginine; Tre, trehalose; Fuc, fucose; Glc: *d-*glucose, Fru: *d-*fructose, Suc, sucrose; Raf, raffinose; Sta, stachyose; Ara, arabinose. N = 5.

**Fig. 1:**
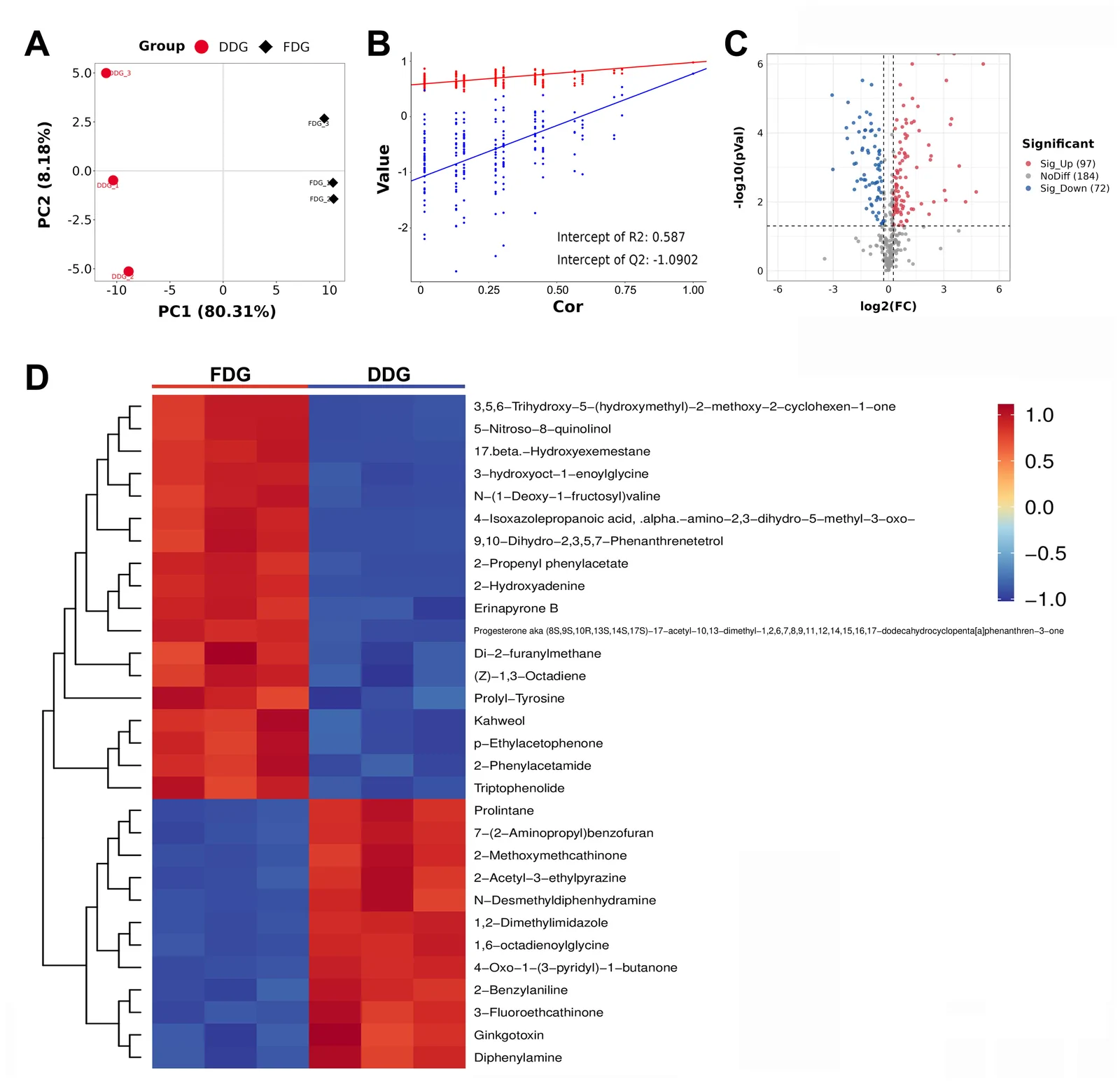
Differential metabolites of DDG and FDG samples assessed using LC-MS. **(A)** Principal component analysis (PCA) of the normalized peak areas of individual metabolites. **(B)** Validation of OPLS-DA results based on 200 permutation tests. OPLS-DA: orthogonal projections to latent structure discriminant analysis; (**C)** Volcano plot of the differences in metabolite abundance between DDG and FDG samples; (**D)** Heat map and hierarchical clustering of differentially expressed metabolites in DDG and FDG samples. Red: upregulated; blue: downregulated. DDG, distiller-dried grains; FDG, fermented distiller-dried grains.

In LC-MS analysis, an overview of the metabolic characteristics of the DDG and FDG samples is shown in Fig. 1. A total of 538 peaks were detected, of which 353 were retained after interquartile-range-based denoising. These were assigned to ten categories, including lipids and lipid-like molecules (45%), organic acids and derivatives (16%), organoheterocyclic compounds (13%), benzenoids (9%), phenylpropanoids and polyketides (5%), organic oxygen compounds (5%), organic nitrogen compounds (2%), alkaloids and derivatives (1%), nucleosides, nucleotides and analogs (1%) and others (2%). PCA showed clear separation of FDG from DDG along PC1 (80.31%) and PC2 (8.18%), indicating distinct metabolic signatures (Fig. 1A). Supervised OPLS-DA analysis yielded R²Y(cum) = 0.999 and Q²Y(cum) = 0.986; 200-permutation tests yielded intercepts of R² = 0.587 and Q² = –1.090, confirming model robustness (Fig. 1B). Differential metabolites (FC > 1.2 or < 0.83, and *P* < 0.05) are shown in a Fig. 1C. A total of 169 metabolites were significantly different, with 97 upregulated and 72 downregulated metabolites in FDG compared with DDG. Their relative abundances are shown in Fig. 1D. Finally, 30 metabolites were prioritized as markers of the main metabolic shifts occurring during fermentation of sorghum distiller’s grain (**Table S1**). Relative to the non-fermented DDG controls, fermented FDG samples exhibited significant upregulation of “Polyphenols and flavonoids” (daidzein and biochanin A), “Nucleosides, nucleotides, and analogs” (adenosine, 2-hydroxyadenine and Leu-Arg), and the majority of “Organic acids and derivatives” [3-hydroxyoct-1-enoylglycine, N-(1-Deoxy-1-fructosy l) valine, and 1-(3-Pyridyl)-1-butanone-4-carboxylic acid] (*P* < 0.05). In contrast, “Alkaloids and derivatives” (tetrahydroharmol, arecaidine, perilline, betaine, and histamine) were significantly downregulated in FDG (*P* < 0.05).

### Apparent digestibility of DDG and FDG diets in vitro and in vivo

As shown in Table 4, the *in vitro* biomimetic digestion assay indicated that fermentation improved the apparent digestibility of DDG samples, increasing apparent dry-matter digestibility (ADMD) from 39.75% to 46.12% and apparent crude-protein digestibility (ACPD) from 44.14% to 61.49%; however, these increments did not reach statistical significance (*P* = 0.180 and *P* = 0.090, respectively). When the same samples were evaluated using the *in vivo* biological method, the values were consistently higher, and the differences between the DDG and FDG samples became more pronounced. ADMD increased from 43.18% to 51.28% (*P* = 0.177), whereas ACPD increased markedly from 46.25% to 71.85% (*P* < 0.01). Apparent ether-extract digestibility remained virtually unchanged (74.25% vs. 75.85%; *P* = 0.494), but apparent crude-fiber digestibility was more than doubled by fermentation (11.91% vs. 22.69%; *P* = 0.026).

**Table 4:** Analysis on the apparent digestibility of DDG and FDG samples in vitro and in vivo.

Items	Groups		SEM	*P* value
	DDG	FDG		
*in vitro* biomimetic digestion method				
ADMD %	39.75	46.12	2.831	0.180
ACPD %	44.14	61.49	3.398	0.090
*in vivo* biological method				
ADMD %	43.18	51.28	7.133	0.177
ACPD %	46.25	71.85	5.585	<0.001
AEED %	74.25	75.85	3.530	0.494
ACFD %	11.91	22.69	3.268	0.026

DDG: distiller’s dried grains; FDG, fermented distiller’s dried grains; ADMD, apparent dry matter digestibility; ACPD, apparent crude protein digestibility; AEED, apparent ether extract digestibility; ACFD, apparent crude fiber digestibility.

### Effects of supplementing different FDG levels on growth performance in broilers

The effects of dietary inclusion of FDG on growth performance are presented in Table 5. During the starter phase (1–21 days), ADFI did not differ among treatments (*P* > 0.05). ADG decreased linearly and quadratically with increasing FDG level (*P* < 0.05); the F20 group had the lowest ADG, which differed significantly from that of the F0 group (*P* < 0.05), whereas ADG in the F2.5–F15 groups was comparable to that of the control. Consequently, F/G was highest in the F20 group and was significantly greater than in the F0 and F2.5 groups (*P* < 0.05), with significant linear and quadratic responses to increasing FDG levels (*P* < 0.05). From day 22 to 42, ADFI was unaffected by FDG levels (*P* > 0.05). The F20 group displayed the poorest F/G (*P* < 0.05), and significant linear and quadratic effects of FDG supplementation on F/G were detected (*P* < 0.05). Over the entire 42-day period, ADFI and ADG did not differ among treatments (*P* > 0.05), but feeding 20% FDG markedly impaired F/G (*P* < 0.01). Increasing FDG levels resulted in linear and quadratic effects in ADG and F/G over the whole period (*P* < 0.01).

**Table 5:** Effects of supplementing different FDG levels on growth performance in broilers.

	Groups						SEM	*P* value		
	F0	F2.5	F5	F10	F15	F20		*P* value	*P* linear	*P* quadratic
1-21 days										
ADFI g/d	41.37	40.03	41.55	41.23	41.98	43.60	0.509	0.510	0.337	0.454
ADG g/d	35.45^a^	34.04^ab^	34.73^a^	33.55^ab^	33.25^ab^	31.67^b^	0.324	0.009	<0.001	0.002
F/G	1.17^a^	1.18^a^	1.20^ab^	1.23^ab^	1.26^ab^	1.38^b^	0.020	0.032	0.002	0.004
22-42 days										
ADFI g/d	139.73	134.22	138.81	136.02	138.89	139.74	0.990	0.641	0.980	0.936
ADG g/d	83.90	83.95	84.63	78.31	81.27	78.77	0.833	0.081	0.001	0.006
F/G	1.63^ab^	1.60^a^	1.64^ab^	1.74^ab^	1.71^ab^	1.77^b^	0.018	0.013	0.002	0.006
1-42 days										
ADFI g/d	89.05	87.13	90.68	88.63	90.44	91.67	0.564	0.762	0.457	0.540
ADG g/d	59.68	59.00	59.68	55.93	57.26	55.22	0.701	0.052	<0.001	<0.001
F/G	1.49^a^	1.48^a^	1.52^a^	1.58^ab^	1.58^ab^	1.66^b^	0.014	0.004	<0.001	<0.001

DDG, distiller’s dried grains; FDG, fermented distiller’s dried grains; ADFI, average daily feed intake; ADG, average daily gain; F/G, ADFI/ADG.

### Effects of supplementing different FDG levels on liver and kidney functions in broilers

The effects of serum indicators on liver and kidney function are presented in Table 6. Dietary FDG supplementation up to 10% did not influence hepatic enzyme activities or creatinine (*P* > 0.05); however, serum uric acid (UA) was significantly reduced at 5% FDG and remained lower at 15% and 20% than in the control (*P* < 0.05). Serum urea nitrogen (UN) tended to decline with increasing FDG levels and was significantly lower in the F20 than in the F0 and F2.5 groups (*P* < 0.05). When the level reached 20%, hepatic alanine aminotransferase (ALT) and aspartate aminotransferase (AST) activities were markedly elevated (P < 0.05) compared with the control, and creatinine (Cr) increased (*P* < 0.05), whereas UN and UA remained significantly lower than the control (*P* < 0.05). Significant linear and quadratic trends were observed for ALT, UN, and UA with increasing FDG levels (*P* < 0.05), whereas only a significant linear trend was detected for Cr (*P* < 0.05) and no significant linear or quadratic trend was detected for AST (*P* > 0.05).

**Table 6:** Effects of supplementing different FDG levels on liver and kidney functions in broilers.

	Groups						SEM	*P* value		
	F0	F2.5	F5	F10	F15	F20		*P* value	*P* linear	*P* quadratic
Liver functions										
ALT U/L	2.67^a^	3.98^ab^	3.87^ab^	3.15^a^	7.08^ab^	8.36^b^	0.560	0.006	0.002	0.005
AST U/L	114.83^a^	138.48^ab^	154.30^ab^	133.11^a^	233.01^a^	483.32^b^	15.922	<0.001	0.059	0.122
Kidney functions										
BUN mg/dL	1.18^bc^	1.30^c^	1.06^bc^	1.02^abc^	0.89^ab^	0.70^a^	0.043	0.015	<0.001	<0.001
Cr μmol/L	13.81^a^	12.80^a^	16.91^ab^	15.46^ab^	17.65^ab^	19.74^b^	0.610	0.004	0.024	0.083
UA umol/L	253.83^b^	147.80^ab^	135.22^a^	201.70^ab^	105.84^a^	126.06^a^	12.800	0.002	0.008	0.007

DDG, distiller’s dried grains; FDG, fermented distiller’s dried grains; ALT, alanine aminotransferase; AST, aspartate aminotransferase; BUN, blood urea nitrogen; Cr, creatinine; UA, uric acid.

### Effects of supplementing different FDG levels on intestinal morphology in broilers

The effects of different FDG supplementation levels on intestinal morphology are shown in Table 7. In the jejunum, villus height showed no differences among treatments (*P* > 0.05), but crypt depth increased linearly with FDG levels and was significantly greater in the F20 than in the F0, F2.5, and F5 groups (*P* = 0.003). The jejunal V/C ratio declined with increasing FDG levels, and F20 exhibited the lowest ratio and differed from those of F0, F2.5, and F5 (*P* = 0.035). Increasing FDG levels linearly and quadratically affected jejunal crypt depth and V/C ratio (*P* < 0.05). In the ileum, villus height (*P* = 0.073) and crypt depth (*P* = 0.183) were not significantly affected by FDG supplementation overall. However, ileal villus height exhibited significant linear and quadratic responses to increasing FDG levels (*P* < 0.05), with the numerically lowest value in the F20 group. The ileal V/C ratio was reduced in F20 (*P* = 0.047) compared with that in the F2.5 and F5 groups, confirming that the morphological alteration occurred only at the highest FDG level. Overall, dietary FDG up to 15% did not significantly alter intestinal architecture, whereas 20% inclusion deepened crypts and lowered the V/C ratio in both jejunum and ileum.

**Table 7:** Effects of supplementing different FDG levels on intestinal morphology in broilers.

	Groups						SEM	*P* value		
	F0	F2.5	F5	F10	F15	F20		*P* value	*P* linear	*P* quadratic
Jejunum										
villus height μm	1281.84	1110.74	1150.33	1308.11	1250.90	1157.77	31.693	0.110	0.620	0.451
crypt depth μm	142.68^ab^	120.89^a^	122.64^a^	151.95^ab^	164.48^bc^	201.00^c^	5.954	0.003	0.004	0.004
V/C	8.98^b^	9.19^b^	9.38^b^	8.61^ab^	7.61^ab^	5.76^a^	0.315	0.035	0.007	0.008
Ileum										
villus height μm	1030.43	1060.33	1043.54	1069.22	1037.00	927.81	15.146	0.073	0.023	0.019
crypt depth μm	163.88	155.31	153.59	174.96	177.93	185.32	4.436	0.183	0.198	0.435
V/C	6.29^ab^	6.83^b^	6.79^b^	6.11^ab^	5.83^ab^	5.01^a^	0.207	0.047	0.035	0.091

DDG, distiller’s dried grains; FDG, fermented distiller’s dried grains; V/C, villus height/crypt depth.

### Effects of supplementing different FDG levels on cecal microbial communities

In the analysis of the cecal microbiota by 16S rRNA (V3-V4) gene sequencing, after quality control and chimera removal, 2,595,083 valid tags were retained, with an average of 74,145 tags per sample (n = 35 samples; one sample was excluded during quality control because of insufficient sequencing depth), which were identified as being of bacterial origin. After dereplication using DADA2, these sequences were assigned to 9489 ASV feature sequences of bacterial species. The key components of biodiversity, including species richness, evenness, and rarity, were measured using Shannon, Simpson, and Chao1 indices (Fig. 2A). The observed species in the F2.5 and F5 groups exhibited higher values than in F20 (*P* < 0.05). The Shannon index of broilers fed the 5% FDG diet was greater than that in the F0 and F20 groups (*P* < 0.05). Addition of FDG to the diet did not affect the Simpson index (*P* > 0.05). The Chao1 indices were higher in the F15 and F20 groups than in the F0 group (*P* < 0.05). Principal coordinate analysis (PCoA) revealed no distinct clustering among the treatment groups along the axes (Fig. 2B). F0, F10, F15, and F20 exhibited relatively high within-group dispersion, whereas F2.5 and F5 clustered intermediately. The overall composition of the microbiota in the cecum at the phylum and genus levels is shown in Fig. 2C. Among these groups, the dominant phyla were Firmicutes, Bacteroidetes, and Proteobacteria, Actinobacteriota and Campylobacterota, and the dominant genera were *Faecalibacterium, Bacteroides, Escherichia-Shigella, Subdoligranulum,* and *Negativibacillus*. Indicator species analysis identified bacterial genera whose abundances were significantly associated with dietary FDG levels (Fig. 2D). Broilers without FDG supplementation were characterized by genera *Escherichia-Shigella, Enterococcus*, and *Tuzzerella* (*P* < 0.05). At the 2.5% FDG level, genera *Intestinimonas, Fournierella*, and *Colidextribacter* displayed higher sqrtIVt values than other genera (*P* < 0.05). Birds receiving 5% FDG were characterized by the genera *Butyricicoccus, Lactobacillus*, and *Faecalibacterium* (*P* < 0.05). The 10% FDG group yielded genera *Eubacterium* and *Shuttleworthia* (*P* < 0.05), whereas 15% FDG produced no indicator genera. At the highest FDG supplementation (20%), *Escherichia–Shigella* emerged as the strongest biomarker (*P* < 0.05).

**Fig. 2:**
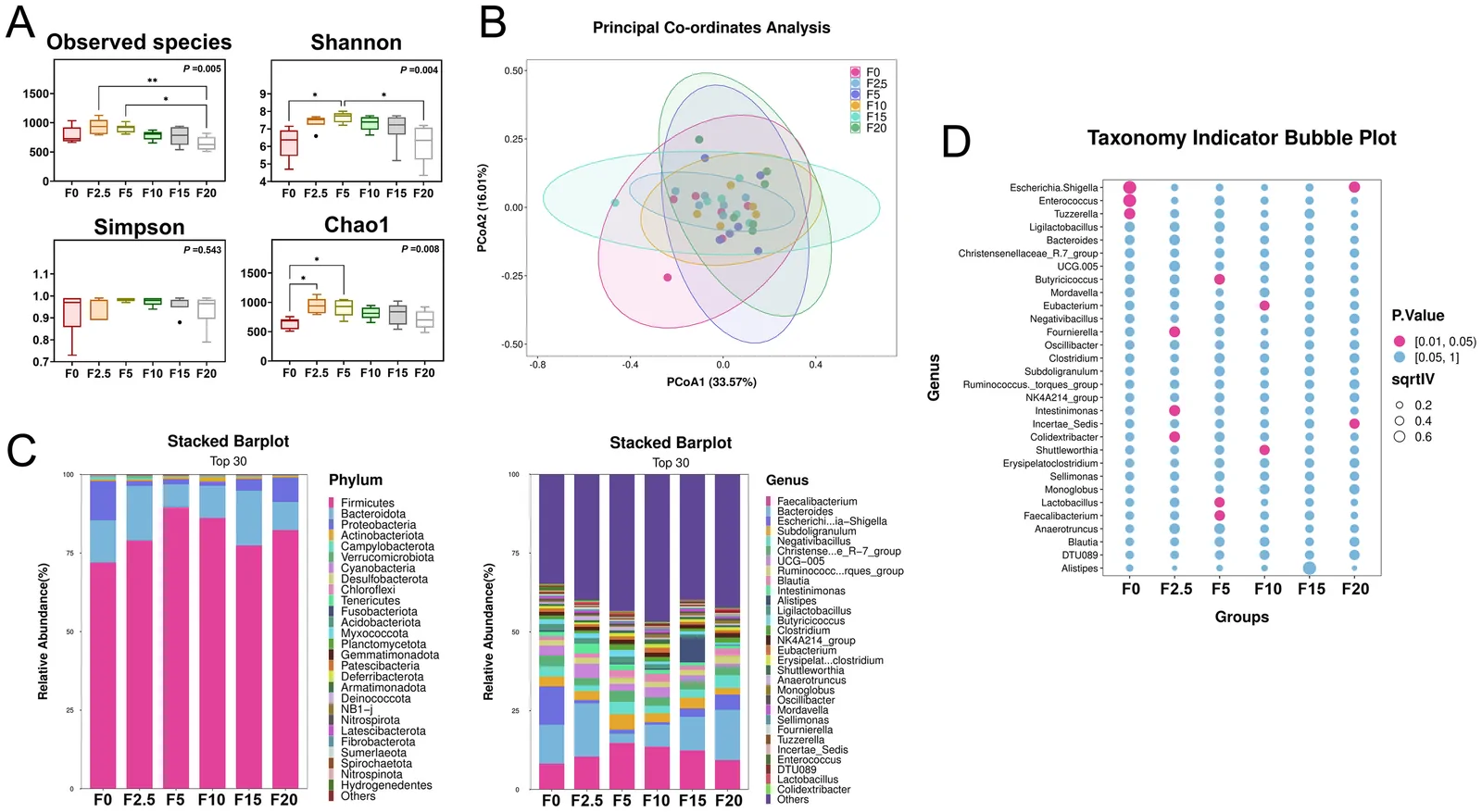
Effects of supplementing different FDG levels on cecal microbial communities in broilers. **(A)** Alpha diversity indices (the observed species, Chao1, Simpson and Shannon indices); **(B)** Principal coordinate analysis (PCoA); **(C)** Overall composition of microbiota and average relative abundance in the experimental groups at phylum and genus levels; **(D)** Indicator species analysis (ISA) base on the average abundances of differential genera. n = 6 replicates.

## Discussion

Numerous studies have claimed that SSF effectively improves the nutritive value of grain processing byproducts, including rice bran (Manlapig et al., 2025), brewers’ spent grain (BSG, Eliopoulos et al., 2022), and flaxseed. Eliopoulos et al. (2022) reported that SSF of BSG resulted in a significant increase in protein content by 49.49% and a respective reduction of cellulose by 11.42%. Similar to their results, SSF increased CP from 17.96% to 21.98% and reduced CF from 19.5% to 14.9%. Although part of these increases may result from the relative concentration of nutrients due to the loss of other organic matter during fermentation, the modest change in crude ash content from DDG to FDG (6.83% vs. 7.46%) indicates that this concentration effect was not the primary cause of the improved nutritional value of FDG. the marked enrichment of microbial metabolites (e.g., nucleosides and polyphenols) in FDG indicates genuine microbial protein synthesis and metabolic conversion.Taken together, although the concentration effect contributed to the elevated nutrient concentrations, microbial fermentation remained the primary reason for the improved feeding value of the distiller’s grains. In the present study, SSF upgraded sorghum-based DDG from a fiber-rich baijiu by-product to a protein feedstuff, which suggests that SSF is a promising bioprocess that enables microorganisms to survive in environments with minimal water (Lock et al., 2024). Additionally, a higher amount of total AAs was due to an increase in 12 of the 17 AAs, especially essential Lys, Met, Thr, Arg, Gly, Ser, Cys, Phe, and His, which closely matched the broiler requirements. This may improve performance and reduce endogenous nitrogen loss through improved protein turnover (Wang et al., 2024). Post-fermentative accumulation of trehalose, glucose, fructose, and the two α-galactosides raffinose and stachyose indicates that both non-digestible polysaccharides hydrolysis and anaerobic metabolism involving microorganism occurred simultaneously (Xie et al., 2024). From a broiler nutrition perspective, the greater content of readily absorbable soluble sugars (e.g., glucose and fructose) and the prebiotic α-galactosides in diets can provide an immediate energy source for intestinal epithelial cells and selectively stimulate probiotics, potentially maintaining intestinal barrier integrity (Kim et al., 2026). Collectively, these changes highlight FDG as a functional protein source capable of partially replacing SBM.

Fermentation-driven metabolite changes endow FDG with more bioactive substances than DDG, which may exert positive physiological functions (Eliopoulos et al., 2022). In this study, daidzein and biochanin A were soy isoflavones that increased 8.59- and 2.54-fold, respectively, after fermentation. It has also been reported that BSG is high in bioactive polyphenols, such as catechin, p-coumaric, and ferulic acid (Eche et al., 2025). These polyphenols act as potent antioxidants and mild phyto-oestrogens that inhibit intestinal inflammation and improve the intestinal health of broiler chickens (Sun et al., 2025; Sarfraz et al., 2020). Concurrently, adenosine and 2-hydroxyadenine increased 4.59- and 6.50-fold, respectively, whereas the dipeptide Leu-Arg increased 2.89-fold, supplying readily absorbable purines and a vasodilatory peptide that enhances cardiac output and nutrient perfusion under heat stress conditions (Chowdhury et al., 2019). Organic acids and derivatives, such as 3-hydroxyoct-1-enoylglycine, N-(1-deoxy-1-fructosy l) valine, and 1-(3-pyridyl)-1-butanone-4-carboxylic acid upregulated, delivering anti-inflammatory cues that can maintain the epithelial barrier and dampen NF-κB signaling in the broiler gut (Kumar et al., 2022; Bortoluzzi et al., 2021). More importantly, fermentation biodegraded alkaloids and derivatives in DDG: tetrahydroharmol, arecaidine, betaine, and histamine decreased 0.65–0.85-fold, thus improving the palatability and reducing the concentrations of anti-nutritional factors in the broiler production.

Changes in the nutrient levels and metabolite composition of DDG also improved digestibility in broilers. In the current study, the CP digestibility *in vitro* increased from 44.14% in DDG to 61.49% in FDG, while that *in vivo* increased from 46.25% in DDG to 71.85% in FDG. Further, the CF digestibility *in vivo* increased from 11.91% for DDG to 22.69% for FDG. This trend of increased digestibility may be related to the degradation of complex macromolecular substances in DDG during SSF (Liu, 2024; Ma et al., 2026). Microorganisms involved in fermentation can secrete a variety of hydrolases, such as cellulase, xylanase, and protease, which can break down cellulose, hemicellulose, and insoluble proteins in DDG into small-molecule substances that are easier to digest and absorb (Swiatkiewicz et al., 2016; Kim et al., 2026), thereby improving the accessibility of nutrients to digestive enzymes, which is consistent with our results on nutritive values between DDG and FDG. Moreover, the gut microbes of broilers may further degrade the undigested protein in the feed, which synergistically promotes the absorption and utilization of proteins (Chen et al., 2025), thus making in vivo digestibility generally higher than in vitro digestibility in our study. Regarding the digestibility of other nutrients, the apparent digestibility of DDG and FDG showed no differences *in vivo*, indicating that fermentation had little effect on the digestibility of fat in DDG. This may be because the EE in DDG is mostly in a comparably readily digestible form, and fermentation does not change its chemical structure or form (Chen et al., 2025).

In the present study, FDG can be incorporated up to 15% without compromising broiler performance, whereas 20% FDG consistently deteriorated feed efficiency across all growth phases, reducing the economic advantage of the cheaper ingredient. VerBeek et al. (2024) also found that including high-protein FDG or corn-fermented protein in poultry diets at 7.5% and 15% had no effect on growth, feed intake, or feed conversion. Thus, FDG can safely replace up to 15% of the conventional corn-SBM diet in broilers. From the perspective of growth performance, the 2.5%–15% FDG range had no adverse effect on ADG or F/G. It was reported that adding 2.5%–5% FDG to the diets increased the ADG of AA broilers and reduced the F/G ratio (Zhang et al., 2018b; Cui et al., 2021). In our study, ADG in 2.5%−5% FDG groups was numerically lower and F/G numerically higher than in the F0 group during days 22–42. Therefore, low-dose FDG (2.5%–5%) exerted no adverse effect on growth performance, and even might be beneficial. In all, FDG can be included up to 15% without compromising performance, and the inclusion level should be selected according to production goals.

Furthermore, the serum biochemistry of liver function provided a clear safety cutoff for FDG supplementation. Our results showed that hepatic transaminases remained stable up to 15% FDG supplementation, but ALT and AST activities increased significantly at the highest level of 20%, indicating potential subclinical hepatocyte damage in broilers (Kaiser et al., 2022). However, it was also reported that plasma AST activity in the 10% and 15% FDG replacement groups decreased in growing-finishing pigs and that FDG supplementation could improve antioxidant abilities of animals (Zhang et al., 2018a), which needed further research. Moreover, kidney function in broilers remained relatively stable from 2.5% to 10% FDG. At the 20% level of FDG, the decrease in BUN and increase in Cr point to a transitional imbalance in renal function (Elhady et al., 2022). Although these changes still stay within a normal physiological range in poultry (Xi et al., 2020), they mark 10% as the practical threshold, above which the combined load of higher fiber, nucleic acids, and alkaloid residues begin to increase the burden on both liver and kidney clearance pathways.

The morphology of intestinal villi reflects the health status of the intestine and its digestive and absorptive capabilities. In our study, the morphology of jejunum and ileum in broilers was relatively complete and healthy when the FDG supplementation under 15%, whereas 20% FDG caused a linear increase in crypt depth and decreased the V/C ratio, suggesting that the integrity of the intestinal mucosa was impaired at high FDG levels. The increase in crypt depth was a compensatory proliferative response of intestinal epithelial cells to the structural damage of the villi (Parsaie et al., 2007). Interestingly, the structural damage to the jejunum was more severe, whereas the ileum, because of its more resilient fermentative epithelium, showed only a modest V/C drop.

Microbiome analysis provides a mechanistic bridge between diet, physiology, and performance. In this study, the low-dose FDG acted as a prebiotic, and the richness and evenness of cecal flora peaked when fed with 2.5%–5% FDG, showing a clear ecological balance. Broilers without FDG supplementation were characterized by genera *Escherichia-Shigella, Enterococcus,* and *Tuzzerella*, which were closely related to the mild inflammation and gut mucosal barrier dysfunction (Qu et al., 2025). Introducing 2.5% FDG displaced the above microbial community characteristics of *Intestinimonas, Fournierella,* and *Colidextribacter*, genera that specialize in fiber-derived aromatic acids and are thought to inhibit pathogen adhesion (Johnson-Henry et al., 2007). At 5% FDG, the community reached its beneficial climax, and *Butyricicoccus, Lactobacillus*, and *Faecalibacterium* dominated, combining butyrate production with competitive exclusion of Enterobacteriaceae (Wang et al., 2021) and coinciding with the highest Shannon diversity and optimal growth performance in the current study. *Butyricicoccus* and *Faecalibacterium*, which are typical butyrate-producing bacteria in the intestinal tract of broilers, can significantly increase the intestinal butyrate content, which can upregulate the expression of tight junction proteins in enterocytes, enhancing the integrity of the intestinal barrier (Shang et al., 2024). *Lactobacillus*, a further dominant genus in the 5% FDG-induced community, exerted a complementary synergistic effect with butyrate-producing bacteria. As a by-product of liquor brewing, FDG undergoes microbial fermentation during production, which enriches it with bioactive substances such as isoflavones, nucleotides, and partial degradation products of cellulose and hemicellulose based on our results. These components directly regulate the abundance of *Butyricicoccus, Lactobacillus, Faecalibacterium*, and Enterobacteriaceae by serving as specific nutrient substrates or signaling molecules for intestinal microorganisms (Iram et al., 2020). Ten percent of FDG were still characterized by *Eubacterium* and *Shuttleworthia*, core Firmicutes genera known to ferment complex polysaccharides; however, the loss of acid-producing probiotics signals the first step toward instability. No characteristic genus was found at 15% FDG, implying a transient, competition-saturated state, whereas 20% FDG reinstated *Escherichia-Shigella* as the leading biomarker in our results. Collectively, 5% FDG supplementation in broiler diets provided the best microbiota richness and beneficial metabolite producers to support maximal broiler performance, whereas the cecal community would slide toward dysbiosis at < 2.5% or > 10%.

## Conclusions

In conclusion, this study systematically evaluated the nutritional value of sorghum-based FDG and its dose-dependent effects on broilers, providing novel insights into the application of fermented baijiu byproducts in poultry nutrition. SSF converts sorghum DDG into a protein-rich, low-fiber ingredient that can safely replace partly corn-SBM diet in broilers. This process increases the CP level and protein digestibility, enriches isoflavones and nucleotides, and degrades alkaloids. FDG supplementation exhibits significant linear and quadratic dose‑response effects on growth performance, serum biochemistry, intestinal morphology, and cecal microflora in broilers. The appropriate FDG inclusion level for growth performance and intestinal health is 2.5%–5%, under which growth performance was comparable to that of the control while intestinal structure and beneficial microbial abundance were improved. For practical soybean meal replacement in commercial production, 10% FDG can be included without compromising production performance and health status, although a formal economic analysis is warranted in the futher study. The maximum safe upper limit of FDG in broiler diets is 15%, while 20% FDG significantly impairs feed efficiency, intestinal morphology, and liver and kidney function, and induces potential dysbiosis of cecal microflora. Collectively, FDG can be used as a high-quality unconventional protein feedstuff in broiler diets, with supplementation levels flexibly selected according to production goals.

## Author contributions

Hailin Huan made substantial contributions to the conception and design of the study, the acquisition and interpretation of the data, and the drafting of the manuscript; Junshu Yan contributed to the acquisition and curation of the data, and to the writing and critical revision of the manuscript; Yuanlu Tu contributed to the acquisition and analysis of the data, and to the critical revision of the manuscript; Kai Zhang contributed to the experimental methodology and sample analyzes, and to the critical revision of the manuscript; Gaiqin Wang contributed to the sample analyzes and data curation, and to the critical revision of the manuscript; Shuaipeng Guo contributed to the 16S rRNA sequencing data analyzes, and to the critical revision of the manuscript; Honghua Huan contributed to the animal experiment and the provision of resources, and to the critical revision of the manuscript; Yumeng Xi made substantial contributions to the conception and design of the study, acquired funding, supervised the project, and critically revised the manuscript. All authors have read and approved the final version of the manuscript and agree to be accountable for all aspects of the work.

## Disclosures

We declare that we have no financial and personal relationships with other people or organizations that can inappropriately influence our work, there is no professional or other personal interest of any nature or kind in any product, service and/or company that could be construed as influencing the position presented in, or the review of, the manuscript entitled, “Evaluation of nutritive value of sorghum-based fermented distiller’s dried grain and its effects on growth performance and intestinal functions in broilers”.
